# SIRT1/2 orchestrate acquisition of DNA methylation and loss of histone H3 activating marks to prevent premature activation of inflammatory genes in macrophages

**DOI:** 10.1093/nar/gkz1127

**Published:** 2019-12-04

**Authors:** Tianlu Li, Antonio Garcia-Gomez, Octavio Morante-Palacios, Laura Ciudad, Sevgi Özkaramehmet, Evelien Van Dijck, Javier Rodríguez-Ubreva, Alejandro Vaquero, Esteban Ballestar

**Affiliations:** 1 Epigenetics and Immune Disease Group, Josep Carreras Leukaemia Research Institute (IJC), 08916 Badalona, Barcelona, Spain; 2 Chromatin and Disease Group, Cancer Epigenetics and Biology Programme (PEBC), Bellvitge Biomedical Research Institute (IDIBELL), 08908 L’Hospitalet de Llobregat, Barcelona, Spain; 3 Chromatin Biology Group, Josep Carreras Leukaemia Research Institute (IJC), 08916 Badalona, Barcelona, Spain

## Abstract

Sirtuins 1 and 2 (SIRT1/2) are two NAD-dependent deacetylases with major roles in inflammation. In addition to deacetylating histones and other proteins, SIRT1/2-mediated regulation is coupled with other epigenetic enzymes. Here, we investigate the links between SIRT1/2 activity and DNA methylation in macrophage differentiation due to their relevance in myeloid cells. SIRT1/2 display drastic upregulation during macrophage differentiation and their inhibition impacts the expression of many inflammation-related genes. In this context, SIRT1/2 inhibition abrogates DNA methylation gains, but does not affect demethylation. Inhibition of hypermethylation occurs at many inflammatory loci, which results in more drastic upregulation of their expression upon macrophage polarization following bacterial lipopolysaccharide (LPS) challenge. SIRT1/2-mediated gains of methylation concur with decreases in activating histone marks, and their inhibition revert these histone marks to resemble an open chromatin. Remarkably, specific inhibition of DNA methyltransferases is sufficient to upregulate inflammatory genes that are maintained in a silent state by SIRT1/2. Both SIRT1 and SIRT2 directly interact with DNMT3B, and their binding to proinflammatory genes is lost upon exposure to LPS or through pharmacological inhibition of their activity. In all, we describe a novel role for SIRT1/2 to restrict premature activation of proinflammatory genes.

## INTRODUCTION

Macrophages (MACs) are required to respond to a wide range of environmental stimuli which specify their functions. Historically classified as both pro-inflammatory and anti-inflammatory, MACs provide versatile and dynamic responses as part of the innate immune system. In order to acquire the corresponding phenotypes of each cell type, MACs undergo very specific changes in gene expression that are mediated by the complex interplay between signalling, transcriptional and epigenetic machineries. Deregulation of these processes results in abnormal MAC function which ultimately forms the basis for many immune diseases.

Sirtuins, highly conserved proteins that belong to the family of class III histone deacetylases, are key regulators of transcriptional and epigenetic landscape. This family of proteins has been implicated in a wide range of biological and pathological processes, including metabolism, aging and inflammation. One important member of the sirtuin family, SIRT1, regulates inflammation in myeloid cells (1,2). Originally reported to deacetylate histones H3 and H4, SIRT1 substrates have now been expanded to several transcription factors (TFs), including the p65 subunit of NF-κB and p53. SIRT1 also determines the epigenetic landscape through interactions with other chromatin-modifying enzymes (3–6). SIRT1 is induced in mature macrophages by anti-inflammatory conditions, such as the exposure to Th2-cytokines and glucocorticoids (7). In fact, SIRT1 has been extensively described to be integral to macrophage biology through several distinct mechanisms. For instance, SIRT1 plays a key role in the self-renewal of murine macrophages through cell cycle and longevity pathways (8). Also, in a murine model of atherosclerosis, *in vivo* treatment with SIRT1-specific inhibitor EX-527 resulted in increased atherosclerotic lesion size through increased intraplaque macrophage infiltration and impaired autophagy (9). Finally, macrophages isolated from SIRT1 transgenic mice exhibited enhanced polarization toward the M2 axis, coupled with decreased expression of TNFα and IL-1β (10).

Another member of the sirtuin family, SIRT2, transiently shuttles to the nucleus during G_2_/M transition and shares redundant roles with SIRT1 in the deacetylation of H4K16 and p65 (11,12). Although less described, SIRT2 also plays a role in macrophage biology, as SIRT2 ameliorates LPS-induced *iNOS* expression in bone marrow macrophages (13) and its activities are required for the hypo-inflammation phase of sepsis in a mouse model (14).

DNA methylation is another crucial regulator of MAC differentiation, and many key genes have been identified to undergo rapid demethylation during terminal myeloid differentiation (15,16), whereas others undergo slower gains of methylation. In addition, key enzymes in maintaining DNA methylation balance, such as DNA methyltransferase 3A (DNMT3A) (17) and Ten-Eleven-Translocation 2 (TET2), are frequently mutated in myeloid leukaemia (18,19), reinforcing the importance of DNA methylation in myeloid differentiation. Furthermore, in specific contexts of terminal differentiation, DNMTs are required to yield the final functional phenotype, as such that downregulation of DNMT3A abolishes immune-suppressive properties of myeloid-derived suppressor cells (20).

In humans, MACs arise from circulating or resident monocytes (MOs) which are largely present in the blood, spleen and bone marrow. MAC differentiation can be achieved *in vitro* by the addition of M-CSF to isolated peripheral blood MOs. M-CSF MACs can be further polarized into a pro-inflammatory or anti-inflammatory phenotype when exposed to lipopolysaccharide (LPS) or IL-4/IL-10 respectively. The plasticity of these MACs render them responsive to further polarization depending on the environmental stimuli encountered, hence they are coined as M0 MACs. Despite the relevance of sirtuins and DNA methylation in myeloid differentiation and function, their interplay has not been explored in this context until now. In the present study, we observed a rapid upregulation of SIRT1/2 proteins upon the addition of M-CSF to MOs to stimulate MAC differentiation, prior to further polarization. We studied the effects of SIRT1/2 inhibition on global gene expression, DNA methylation and changes in various histone marks during macrophage differentiation and activation. In brief, we report a novel role for SIRT1/2 in the sequential and hierarchical deposition of DNA methylation, involving a direct interaction with DNMT3B, and the inhibition of active histone H3 modifications in pro-inflammatory genes to prevent their premature activation during differentiation and prior to the encounter with bacterial antigens.

## MATERIALS AND METHODS

### MO isolation, differentiation and culture

Buffy coats used in the study were obtained from anonymous donors via the Catalan Blood and Tissue Bank (Banc de Sang i Teixits; BST). All donors signed an informed consent form prior to donation, and all donations were in accordance with the guidelines of the World Medical Association (WMA) Declaration of Helsinki. Peripheral blood mononuclear cells (PBMCs) were extracted by layering buffy coats on Lymphocytes Isolation Solution (Rafer, Zaragoza, Spain) and centrifuged at 800 g for 30 min in the absence of braking. After collection, PBMCs were washed with phosphate-buffered saline (PBS) and MOs were purified using CD14 MicroBeads (Miltenyi Biotec, Bergisch Gladbach, Germany) following the manufacturers’ instructions. Following cell separation, CD14+ MOs were attached to plates by incubation with serum-free medium and then cultured in α-minimal essential medium (α-MEM; Invitrogen, Carlsbad, CA, USA) containing 10% fetal bovine serum, 1% Penicillin Streptomycin (Gibco Thermo Fisher Scientific, MA, USA) and supplemented with 25 ng/ml human M-CSF (PeproTech EC, London, UF) for MAC differentiation. For MAC activation, 10 ng/ml of LPS (from *E. coli* O111:B4, Sigma-Aldrich, Darmstadt, Germany) were added at day 5 of differentiation for 18 hrs. To inhibit SIRT1, SIRT2 and in combination, 70 μM of EX-527 (Sigma-Aldrich, Missouri, USA), 4 μM of AGK2 (Sigma-Aldrich) and 50 μM of Cambinol (BioVision, California, USA) were added, respectively, to MOs at day 0 in the presence of M-CSF. Cytokines and inhibitors were refreshed every two days until the end of the experiment. Corresponding volumes of DMSO were used as control.

### Flow cytometry analysis

Cells were detached mechanically using cell scrapers and resuspended in staining buffer (PBS containing 2 mM of EDTA and 4% FBS), followed by incubation with human FcR Blocking Reagent (Miltenyi Biotec). Cells were counted and 1 × 10^5^ cells were stained with fluorochrom-conjugated antibodies against CD163-FITC (BioLegend, California, USA), CD86-APC (Miltenyi Biotec), CD83-APC (Miltenyi Biotec) and CD80-PE (Miltenyi Biotec) for 20 min on ice protected from light. Cells were washed twice with staining buffer and fixed with PBS containing 4% paraformaldehyde (Electron Microscopy Sciences, PA, USA). 8000 events were acquired using Gallios Flow Cytometer (Beckman Coulter, CA, USA) and analyzed using the Kaluza software (Backman Coulter).

### Quantitative real-time PCR

Total RNA was isolated utilizing Maxwell^®^ RSC simplyRNA Cells Kit (Promega, Wisconsin, USA) and reverse transcription was performed using Transcriptor First Strand cDNA Synthesis Kit (Roche, Basel, Switzerland) all according to manufacturers’ instructions. LightCycler^®^ 480 SYBR Green I Master mix (Roche) and forward and reverse primers (sequences annotated in Supplementary Table S1) were added to the cDNA. Quantitative PCR was carried out using LightCycler^®^ 480 II System (Roche) and results were analyzed using the corresponding LightCycler^®^ 480 Software (Roche). Relative quantification of target genes was calculated by the standard double delta Ct method, and all genes were normalized against the housekeeping gene ribosomal protein L38 (*RPL38*). All statistical tests were performed using ΔCt values in which statistical significance was defined as *P* < 0.05 by paired Student's *t*-test.

### Western blotting

Protein expression was visualized by western blotting, in which electrophoresis and transfer were performed as previously described (16). Membranes were then incubated with primary antibodies against SIRT1 (07-131, Millipore), SIRT2 (ab51023, Abcam), NF-κB p65 (sc-372, Santa Cruz Biotechnologies, TX, USA), p65K310ac (ab19840, Abcam, Cambridge, UK), DNMT1 (NB100-56519, Novus), DNMT3A (3598, Cell Signaling), DNMT3B (sc-20704, Santa Cruz) and β-Actin (ab6276; Abcam). HRP-conjugated mouse and rabbit secondary antibodies were from Thermo Fisher (Massachusetts, USA) and Abcam respectively. Membranes were developed by Amersham™ ECL™ Western Blotting Detection Reagents (GE Healthcare, Illinois, USA) and signals were detected by Amersham™ Imager 600 (GE Healthcare). Band intensities were quantified using the Fiji Software, and relative protein expression was calculated by normalizing against β-Actin expression.

### Enzyme-linked immunosorbent assay (ELISA)

Released cytokines IL-10 and IL-1β were detected using ELISA kits provided by BioLegend and Invitrogen (California, USA), respectively, according to manufacturers’ instructions. Absorbance was measured at 450 nm using PowerWave™ XS microplate reader (BioTek^®^ Instruments, Vermont, USA).

### Chromatin immunoprecipitation (ChIP) assays

ChIP assays were performed as previously described (16). Briefly, MOs, DMSO- and cambinol-treated day 5 and LPS-stimulated MACs were crosslinked with 1% methanol-free formaldehyde (Thermo Fisher) for 15 min and subjected to immunoprecipitation after sonication. ChIP experiments were performed using the LowCell# ChIP kit™ protein A (Diagenode, Liège, Belgium). We used antibodies against acetylated lysine 16 of histone H4 (H4K16ac; 07-329, Millipore), monomethylated lysine 4 of histone H3 (H3K4me1; ab8895, Abcam), trimethylated lysine 4 of histone H3 (H3K4me3; CS200580, Millipore), acetylated lysine 27 of histone H3 (H3K27ac; 07-360 Millipore), SIRT1 (07-131, Millipore), SIRT2 (ab51023, Abcam), PU.1 (PA5-17505, Invitrogen) and DNMT3B (sc-20704, Santa Cruz). Corresponding rabbit IgG (Diagenode) is used as control. Protein binding was analyzed by real-time quantitative PCR, and data are represented as ratio of the enriched fraction with respect to input. ChIP primers were designed for the areas flanking differentially methylated CpGs and their sequences are shown in Supplementary Table S1.

### Co-immunoprecipitation (Co-IP) assays

Co-IP assays were performed using M-CSF macrophages differentiated from CD14+ monocytes for 5 days. Cell extracts were prepared in lysis buffer [50 mM Tris–HCl, pH 7.5, 1 mM EDTA, 150 mM NaCl, 1% Triton-X-100, protease inhibitor cocktail (cOmplete™, Merck)] with corresponding units of Benzonase (Sigma) and incubated at 4°C for 4 h. 50 μl of supernatant was saved as input and diluted with 2× Laemmli sample buffer (4% SDS, 20% glycerol, 120 mM Tris–HCl, pH 6.8). Supernatant was first incubated with PureProteome™ Protein A/G Mix Magnetic Beads (Millipore) for 1 h to remove background signal. Samples then incubated overnight at 4°C with corresponding antibodies against DNMT1 (NB100-56519, Novus), DNMT3A (3598, Cell Signaling), DNMT3B (sc-20704, Santa Cruz), SIRT1 (07-131, Millipore), and SIRT2 (ab51023, Abcam) according to the specifications of each antibody. Negative controls were incubated with rabbit (12-370; Merck Millipore) and mouse (12-371; Merck Millipore) IgGs. Subsequently, samples were incubated with magnetic beads at 4°C for 2 h, and beads were then washed three times with lysis buffer. For sample elution, 100 μl of 1× Laemmli sample buffer was added to beads. Samples and inputs were denatured at 95°C in the presence of 1% β-mercaptoethanol. Whole-cell extracts and IP samples were visualized by western blotting, as described above.

### Transfection of primary human MOs

For RNA knockdown experiments, we transfected isolated monocytes with SIRT1 Silencer® pre-designed siRNA (AM16708-136457, Ambion, TX, USA), and ON-TARGETplus SMARTpool siRNAs against SIRT2 (L-004826-00), DNMT3A (L-006672-01) and DNMT3B (L-006395-00), with non-targeting siRNA as control, all provided by Dharmacon (Colorado, USA). Transfections were carried in MAC differentiation media using Lipofectamine 3000 Reagent (Thermo Fisher Scientific Co., Carlsbad, CA, USA) according to manufacturer's instructions. Media containing siRNA and Lipofectamine were removed after 6–8 h following transfections, and this was repeated every 48 h in M-CSF differentiation media for 5 days, in which MACs were subsequently stimulated with LPS for 18 h. Transfection efficiencies were examined by western blotting.

### Bisulfite modification and pyrosequencing

Total DNA was extracted by the proteinase K protocol. Briefly, cells were lysed using lysis buffer (50 mM Tris pH 8.8, 10 mM EDTA pH 8.3, 100 mM NaCl, 1% SDS) in the presence of Proteinase K (Roche), and nucleic acids and lipids were separated by repeated centrifugation. DNA was precipitated using isopropanol and washed with 75% ethanol. 300 μg of isolated DNA were bisulfite (BS)-converted using EZ DNA Methylation-Gold™ Kit (Zymo Research, CA, USA) according to manufacturers’ instructions. BS-converted DNA (∼20 ng) was used as template for further pyrosequencing. Primers for PCR amplification and sequencing were designed using the PyroMark^®^ Assay Design Software 2.0 (QIAGEN, Hilden, Germany) and sequences are listed in Supplementary Table S1. Conventional PCR was carried out using IMMOLASE™ DNA Polymerase kit (Bioline, London, UK) and PCR products were pyrosequenced with PyroMark^®^ Q24 System (QIAGEN).

### Gene expression array and analysis

The quality of extracted total RNA was first analyzed by the 2100 Bioanalyzer System (Agilent, CA, USA), and 100 ng of excellent quality RNA (RNA Integrity number of > 9) was then hybridized on Human Clariom S™ Assay arrays (Thermo Fisher) carried out at/by the High Technology Unit (UAT), at Vall d’Hebron Research Institute (VHIR) following manufacturers’ instructions. Downstream data normalization and analysis were performed using statistical analysis language R in combination with Bioconductor (https://www.bioconductor.org) and CRAN (https://cran.r-project.org) repository packages. Firstly, background correction was performed using Robust Microarray Analysis (RMA) normalization provided by oligo package. Each probe was then annotated using clariomshumantranscriptcluster.db package, and the average expression level was calculated if more than one probe was mapped to the same gene. Downstream statistical analyses were performed using an eBayes-moderated paired *t*-test provided by limma package to obtain log_2_ fold change (log_2_FC), *P*-value and adjusted *P*-value (Benjamini Hochberg-calculated FDR) between sample groups. Genes that displayed statistically significant tests (*P*-value < 0.01 and FDR < 0.05) were considered differentially expressed. Heatmaps were generated using calculated z-scores.

### DNA methylation profiling and analysis

500 ng of BS-converted DNA were hybridized on Infinium MethylationEPIC BeadChip array (Illumina, Inc., San Diego, CA, USA) following manufacturers’ instructions. This array covers over 850 000 (850K) CpG methylation sites per sample at single-nucleotide resolution, covering 99% of RefSeq genes. Probe fluorescence was detected by BeadArray Reader (Illumina, Inc.), and image processing and intensity data extraction were performed as previously described (16). For downstream normalization, filtering and analysis, standard pipelines from minfi and limma packages were used and data was processed using statistical analysis language R. Probes were filtered by detection *P*-value, in which a *P*-value < 0.01 was considered trustworthy. Background correction was carried by quantile normalization and CpGs in SNPs were excluded. M and beta values were calculated using minfi, and M values were subsequently used for downstream statistical analysis, using the limma package, to obtain *P*-value and adjusted *P*-value (Benjamini–Hochberg-calculated FDR) between sample groups by an eBayes-moderated paired *t*-test. *P*-value of < 0.01 and FDR of < 0.05 was considered statistically significant.

### Gene ontology, motif and pathway enrichment analyses

Gene ontology (GO) analysis of DNA methylation was carried out using GREAT online tool (http://great.stanford.edu/public/html) (21) by applying the basal plus extension settings to annotate genomic regions. Annotated CpGs in the EPIC array were used as background, and GO terms with *P*-value < 0.01 and FC > 2 were considered significantly enriched. GO analysis of gene expression was carried out using the online tool DAVID (https://david.ncifcrf.gov) and annotated genes in the Clariom S array were used as background. The Functional Annotation tool was used and GO terms with *P*-value < 0.01 was considered significantly enriched.

Motif enrichment analyses were performed using HOMER motif discovery software (22) and analyses were carried out using the statistical programming language Unix. Annotated CpGs in EPIC array and genes in Clariom S array were used as background for methylation and expression analyses respectively. For DNA methylation, a window of ±250 bp around the interrogated CpGs was applied, and for gene expression, a window of –2000 bp upstream of the TSSs of interrogated genes was applied. A *P*-value < 0.01 defines a motif as significantly enriched.

Pathway enrichment analyses of gene expression were carried out using GSEA (Gene Set Enrichment Analysis) software. Genes were ranked by expression log_2_FC of Cambinol-treated versus untreated M-CSF MACs, and a Normalized Enrichment Score (NES) was calculated for each hallmark as described in the original article (23).

### Analyses of DNase I hypersensitivity and ChIP-seq of histone modifications

DNase I hypersensitivity and ChIP-seq data of histone modifications H3K27ac, H3K4me1, H3K4me3, H3K27me3, H3K36me3 and H3K9/14ac of MO and MACs were downloaded from the BLUEPRINT portal (http://dcc.blueprint-epigenome.eu/). More than three independent datasets were downloaded for each histone mark and ChIP-seq peaks were consolidated, filtering the peaks with a *q*-value < 0.01 and a fold change ≥2 and using the MSPC program (24) with the parameters -w = 1E^−4^, -s = 1E^−8^, and -c = 3.

## RESULTS

### SIRT1/2 become rapidly upregulated during macrophage differentiation and their inhibition upregulates many inflammation-related genes

**Figure 1.**
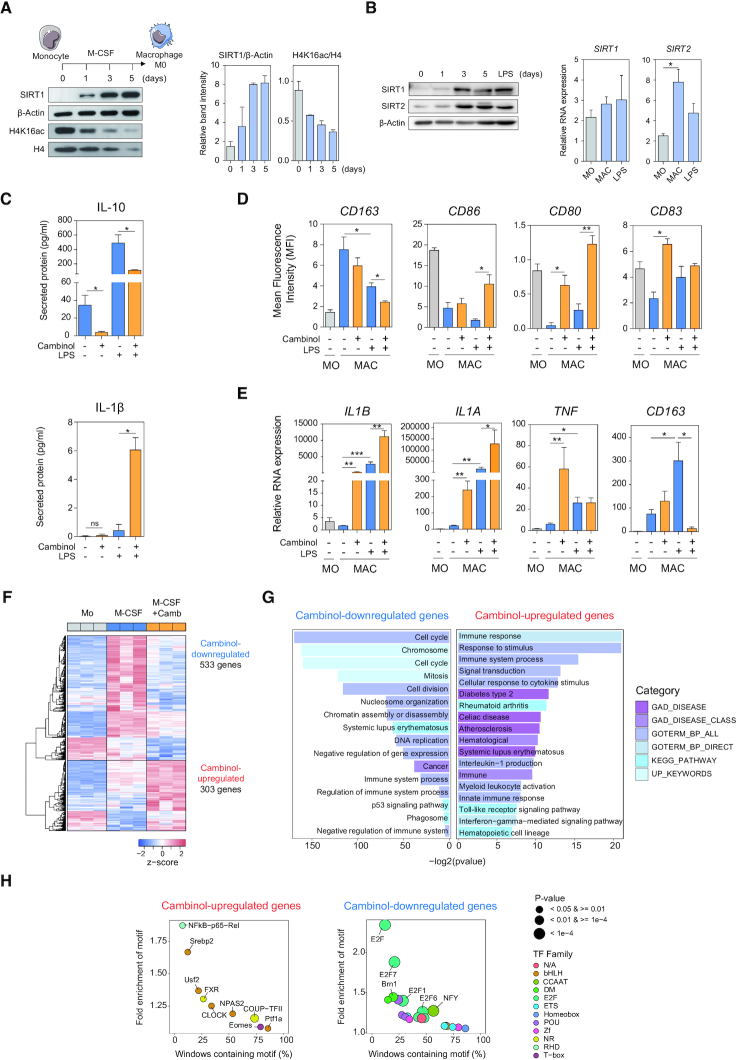
Inhibition of SIRT1/2 with cambinol during MO-to-MAC differentiation results in an aberrant pro-inflammatory phenotype. (A) A representative western blotting analysis showing SIRT1 and acetylated lysine 16 of histone H4 (H4K16ac) protein expression of peripheral blood CD14+ MOs stimulated with 25 ng/mL of M-CSF for 0, 1, 3 and 5 days. Relative band intensities of SIRT1 and H4K16ac were normalised against β-Actin and H4 respectively (right panel) from two independent experiments. (B) Western blot analysis of protein expression of SIRT1 and SIRT2 in MO exposed to M-CSF for 0, 1, 3 and 5 days and activated by the addition of LPS for 18 hrs. RNA expressions of *SIRT1* and *SIRT2* were normalised against *RPL38*. (C) Secreted levels of IL-10 and IL-1β were analysed by ELISA in MACs differentiated in the presence of DMSO or treated with 50 nM of cambinol. (D) FACS analysis of surface markers CD163 (FITC), CD86 (APC), CD80 (PE) and CD83 (APC) in MOs and M-CSF MACs before and after treatment with LPS for 18 hrs in the presence and absence of cambinol (50 nM). (E) Gene expression analysis of *IL1B*, *IL1A*,*TNF* and *CD163* in MOs, cambinol- or DMSO-treated M-CSF MACs differentiated for 5 days and MACs treated with LPS for an additional 18 hrs. (A-E) Statistical significance of at least three independent experiments was calculated by paired student t-test (* p-value < 0.05, ** p-value < 0.01 and *** p-value < 0.001). (F) Heatmap representation of z-scores obtained from log2 expression of genes that undergo significant changes (FDR < 0.05) in cambinol-treated (M-CSF + Camb) compared to DMSO-treated (M-CSF) MACs, in which 533 and 303 genes become downregulated (log2FC < 0 and FDR < 0.05) and upregulated (log2FC > 0 and FDR < 0.05) respectively. (G) GO analyses of cambinol-downregulated (left) and -upregulated (right panel) genes using the DAVID tool (https://david.ncifcrf.gov). (H) Bubble plot representation of HOMER TF motif enrichment analysis. A window of -2000 bp upstream of TSS was used for all genes analysed.

MOs isolated from PBMCs by CD14 positive selection were differentiated *in vitro* into MACs by the addition of M-CSF for 5 days (16). In the absence of further stimuli, these MACs retain a plastic phenotype, in which the cells are susceptible to polarization to either an inflammatory (M1) or anti-inflammatory (M2) phenotype through the addition of LPS or IL-10/IL-4 respectively (25–27). We observed an increase of the SIRT1 protein levels after 24 h following M-CSF addition that continued during the 5 following days, suggesting an essential role of this deacetylase in M-CSF MAC differentiation (Figure 1A). SIRT1 upregulation was concomitant with a decrease in its substrate H4K16Ac (Figure 1A). The upregulation of SIRT1 occurred in parallel with SIRT2 (Figure 1B). The two sirtuins did not experience further changes in their protein levels following LPS stimulation (Figure 1B). *SIRT1* mRNA levels remained relatively stable during this process (Figure 1B), indicating that the increase at the protein level was due to post-transcriptional mechanisms. Contrastingly, *SIRT2* RNA became significantly upregulated during MO-to-MAC differentiation in parallel to its protein levels (Figure 1B). The rapid upregulation of these sirtuins during MAC differentiation are consistent with their described roles in MAC biology, as well as suggesting a potential role during MAC differentiation.

Subsequently, we inhibited SIRT1/2 activity utilizing cambinol, and inhibition efficiency was ascertained by the increase in acetylated p65, a target of SIRT1/2 (4), in cambinol-treated MACs (Supplementary Figure S1A). Consequently, we observed that cambinol-treated MACs underwent various phenotypic changes, including a decrease in the release of anti-inflammatory cytokine IL-10 both before and after LPS stimulation, and increased release of IL-1β after activation (Figure 1C). We also observed that cambinol treatment resulted in the downregulation of the macrophage scavenger receptor CD163 and upregulation of inflammatory surface markers including CD86, CD80 and CD83 (Figure 1D). Further gene expression analysis revealed that upon cambinol treatment, MACs upregulated such proinflammatory genes as *IL1B*, *IL1A* and *TNF* before and in some cases after LPS activation, and downregulated *CD163* expression after LPS treatment (Figure 1E). Analyses utilizing specific inhibitors against SIRT1 (EX527) and SIRT2 (AGK2) suggested a stronger effect upon SIRT1 inhibition compared to SIRT2 inhibition (Supplementary Figure S1B); however, both inhibitors, even in combination, yielded weaker effects than cambinol.

To better characterize the impact of SIRT1/2 inhibition, we hybridized expression microarrays with MOs, M-CSF MACs and cambinol-treated M-CSF MACs and observed that cambinol treatment during MO-to-MAC differentiation resulted in the significant (FDR < 0.05) upregulation and downregulation of 303 and 533 genes respectively (Figure 1F). More specifically, many genes associated with the inflammatory niche were transcriptionally upregulated when SIRT1/2 was inhibited (Supplementary Table S2). Further gene ontology analyses revealed that upregulated genes were enriched in categories including immune response, myeloid activation, and Toll-like receptor and IL-6 signalling. Regarding downregulated genes, we observed enrichment in categories such as cell cycle, DNA replication, nucleosome organization, and E2F targets (Figure 1G, Supplementary Figure S1C). TF motif enrichment analyses revealed the participation of factors such as NF-kB, which is a known target of SIRT1/2 and relevant to inflammation, in the set of upregulated genes (Figure 1H).

Altogether, SIRT1/2 inhibition during macrophage differentiation resulted in the acquisition of a more pro-inflammatory signature, suggesting a role for these deacetylases in the silencing of inflammatory genes prior to MAC polarization.

### SIRT1/2 activity is dispensable for active demethylation but determines *de novo* gains of DNA methylation during macrophage differentiation

**Figure 2.**
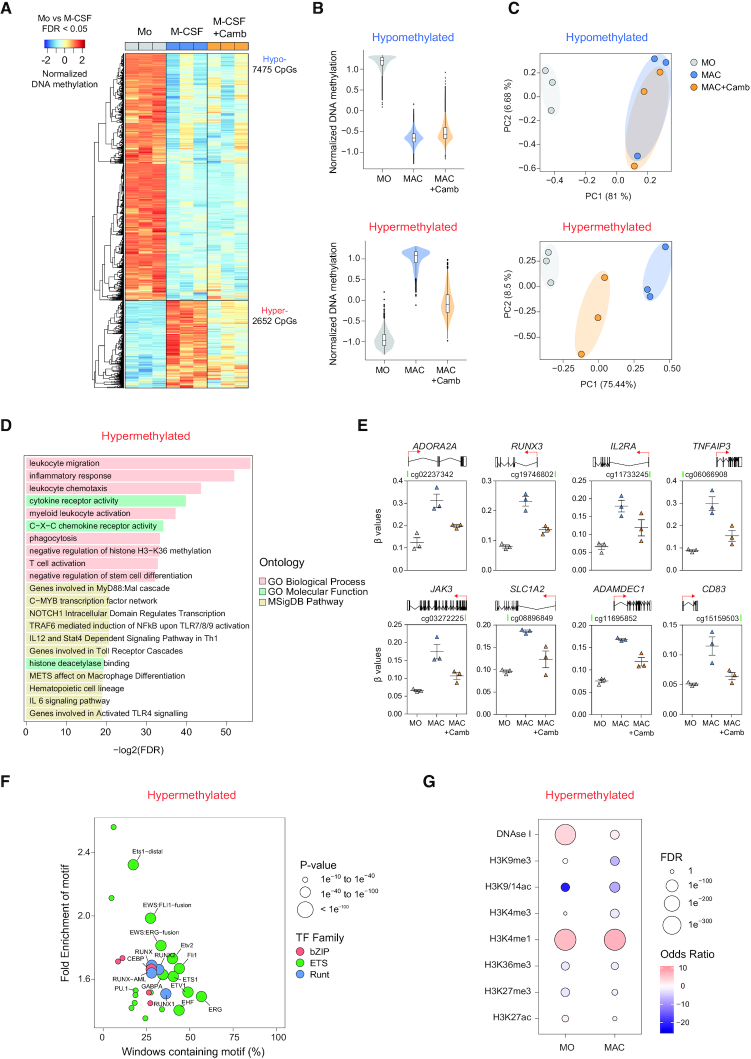
Inhibition of SIRT1/2 inhibition with cambinol affects DNA hypermethylation during MO-to-MAC differentiation. (A) Heatmap representation of significant (FDR < 0.05) DNA methylation changes during MO-to-MAC differentiation comparing DMSO-treated MACs (M-CSF) with MOs. (B) Violin plots depicting the distribution and probability density of z-scores of hypo- (upper) and hypermethylated (lower panel) CpGs. (C) Principal component analysis (PCA) of hypo- and hypermethylated CpGs of the three cell populations (Mo, M-CSF and M-CSF + Camb). (D) GO analysis of hypermethylated CpGs, in which CpGs were mapped to genes using GREAT online tool (http://great.stanford.edu/public/html) by applying the basal plus extension settings, using EPIC array as background. (E) Representation of the beta values of selected hypermethylated CpGs that map to relevant genes. (F) HOMER TF motif enrichment analysis of hypermethylated CpGs were carried out using a window of ±250 bp centring around the CpGs. CpGs annotated in the EPIC 850K array were used as background. (G) Histone marks enrichment analysis of hypermethylated CpGs were carried out by crossing DNase-seq and ChIP-seq data of H3K9me3, H3K9/14ac, H3K4me3, H3K4me1, H3K36me3, H3K27me3 and H3K27ac of MOs and MACs downloaded from the Blueprint database (refer to Materials and Methods for data processing).

MO to MAC differentiation is accompanied by large-scale DNA methylation changes, both gains and losses of methylation, which are critical to the acquisition of the final phenotype of MACs (16,28,29). Given that SIRT1/2 participates in macrophage differentiation and their inhibition results in broad changes in gene expression, we investigated their potential interplay with DNA methylation changes. Utilizing high density bead arrays, we observed that 7475 CpGs became demethylated (FDR < 0.05; hypomethylated cluster) upon MAC differentiation from MO, and this demethylation was not affected when SIRT1/2 were inhibited by cambinol, suggesting that they do not participate in active demethylation (Figure 2A and B, top). The hypomethylated cluster included CpGs of genes associated with innate responses, such as *TM7SF4*, *ACP5* and *ADAM12*, and displayed enrichment in motifs of the bZIP family of TFs, in concordance to a previous study (Supplementary Figure S2B-D) (16). Conversely, 2652 CpGs gained *de novo* methylation (FDR < 0.05; hypermethylated cluster) during MAC differentiation, and surprisingly, inhibition of SIRT1/2 in turn inhibited almost all CpG methylation gains, which suggested that SIRT1/2 activity is required for MAC-associated DNA hypermethylation (Figure 2A and B, bottom). Analyzing variance between samples using principal component analysis (PCA), we showed that for demethylated CpGs, MACs and cambinol-treated MACs displayed overlapping PC dimensions. In contrast, for hypermethylated CpGs, the three cell populations separated along the axis of PC1, in which cambinol-treated MACs showed less PC1 variance with MOs than MACs (Figure 2C). Further analyses of CpG positioning revealed that both hyper- and hypomethylated CpGs were enriched in open seas with a slight enrichment in 5′-UTR compared to background (Supplementary Figure S2A). GO analysis revealed that most of the genes associated with hypermethylated CpGs were enriched in functional categories related to macrophage biology including inflammatory response, cytokine receptor activity and leukocyte migration, and these genes include *ADORA2A*, *RUNX3*, *IL2RA*, *TNFAIP3*, *JAK3*, *SLC1A2*, *ADAMDEC1* and *CD83* (Figure 2D and E). These results suggested that gains of methylation at genes associated with these biological processes were altered when SIRT1/2 were inhibited. Inspection of the protein levels of DNMTs revealed a significant downregulation of DNMT3A protein in cambinol-treated MACs, with both DNMT1 and DNMT3B displaying similar trends without reaching statistical significance (Supplementary Figure S2E).

Sequences associated with hypermethylated CpGs were enriched in a distinct set of TFs, namely members of the ETS family (Figure 2F). Previously, PU.1 and other members of the ETS TF family was linked to hypermethylated regions in terminal myeloid differentiation model, in which PU.1 was observed to recruit DNMT3B to mediate *de novo* DNA methylation (30). Therefore, given these results, it is conceivable that SIRT1/2 activity is required for ETS TF-mediated recruitment of DNMTs to hypermethylated CpG sites.

SIRT1 determines the epigenetic landscape not only through its ability to directly deacetylate H1, H3 and H4 (31–33), but also through the post-translational regulation of other histone-modifying enzymes (34). Given the role of SIRT1 in histone modifications and its effect on DNA methylation, we then analyzed the enrichment of various histone marks in both MOs and MACs, obtained from publically available Blueprint ChIP-seq data (http://dcc.blueprint-epigenome.eu; refer to Materials and Methods for the consolidation of data), in the hypermethylated CpG cluster. We observed that there was a significant enrichment in DNase I hypersensitivity in MOs which decreased in MACs, coupled with significant enrichment of H3K4me1 in both cell types (Figure 2G). These observations were in accordance to what has been previously described in regards to the relationship between DNA methylation and chromatin states, in which gains of DNA methylation in regions of enhancers correspond to heterochromatin (35,36).

### SIRT1/2 activity is required to prevent premature expression of pro-inflammatory genes upon LPS stimulation through premeditated DNA hypermethylation

**Figure 3.**
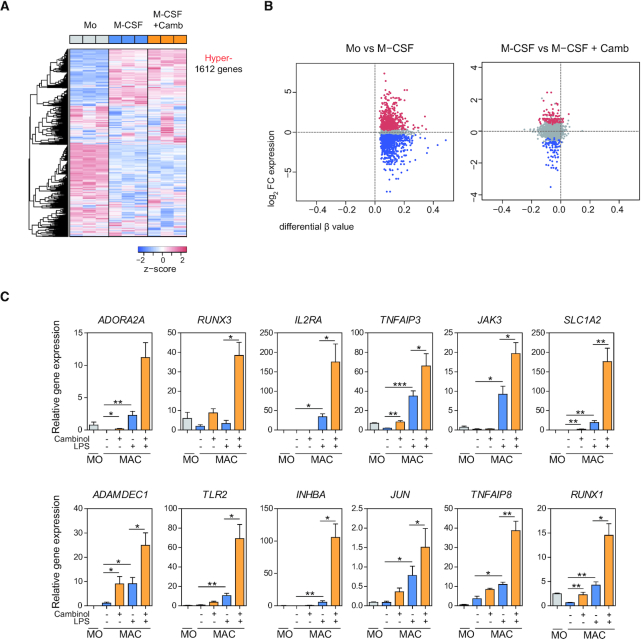
Treatment with cambinol upregulates pro-inflammatory genes associated to hypermethylated CpGs following MAC activation with LPS. (**A**) Color-coded heatmap representation of gene expression of hypermethylated CpGs of the three cell populations (MO, M-CSF and M-CSF + Camb). 2652 hypermethylated CpGs were mapped to 1612 unique genes utilizing GREAT online tool with the setting of mapping to the nearest gene. (**B**) Scatter plots representing log_2_FC of gene expression (y-axis) and difference in beta (Δbeta; x-axis) of M-CSF in respect to MOs (left panel) and M-CSF + Camb in respect to M-CSF MACs (right panel). Genes that show significant (FDR < 0.05) upregulation or downregulation are depicted in red and blue respectively. (**C**) Gene expression analyses of *ADORA2A*, *RUNX3*, *IL2RA*, *TNFAIP3*, *JAK3*, *SLC1A2*, *ADAMDEC1*, *TLR2*, *INHBA*, *JUN*, *TNFAIP8*, *RUNX1* in MOs, MACs and LPS-activated MACs in the presence and absence of 50 μM cambinol. Bar graphs represent mean and standard deviation of relative expression of each gene of four independent experiments normalized against *RPL38*. Statistical significance was calculated using paired Student's *t*-tests (*P-value < 0.05, **P-value < 0.01 and ***P-value < 0.001).

The relationship between DNA methylation and gene expression has been nothing short of controversial. It is widely accepted that DNA methylation inhibits gene expression, however mounting evidence suggest a more complicated reciprocal relationship (reviewed by Jones *et al.* (37)). To evaluate the relationship between gene expression and DNA methylation in relation to SIRT1/2 inhibition, we first mapped the hypermethylated CpGs to the nearest gene, yielding 1612 unique genes (Figure 3A). Surprisingly, only 63 genes (3.9%) displayed upregulation and 61 genes (3.8%) displayed downregulation mediated by cambinol treatment, whereas 92.3% of genes showed no significant gene expression changes despite the inhibition of DNA hypermethylation (Figure 3B, right panel). In contrast, majority of genes from the hypermethylated CpG cluster did display a significant change in expression during the MO-to-MAC differentiation step; however, both upregulation and downregulation were observed (Figure 3A, left panel), which emphasized the complex relationship between DNA methylation and gene expression. Since the pro-inflammatory phenotype observed in cambinol-exposed MACs appeared more pronounced following LPS-mediated activation (Figure 1C–E), it is therefore of interest to interrogate the expression of genes associated to hypermethylated CpGs following LPS stimulation. Interestingly, upon LPS-stimulation, genes such as *ADORA2A, RUNX3, IL2RA*, *TNFAIP3*, *JAK3*, *SLC1A2*, *ADAMDEC1*, *TLR2*, *INHBA*, *JUN*, *TNFAIP8*, *RUNX1*, previously described to be upregulated upon LPS stimulation (38), displayed further upregulation in cells exposed to cambinol (Figure 3C), indicating that SIRT1/2 determined, at least in part, their repression, and DNA methylation might participate in tight control of their repressive status prior to polarization to a pro-inflammatory phenotype.

### SIRT1/2 play concurrent roles in *de novo* gains of DNA methylation and histone modifications in macrophage differentiation and activation

**Figure 4.**
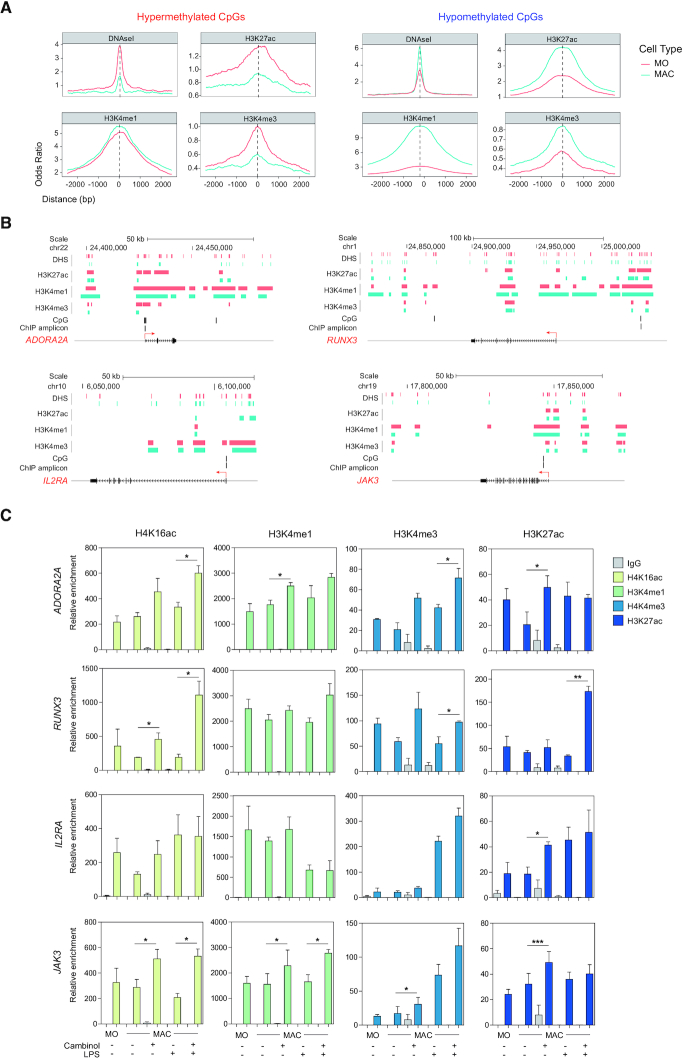
MO-to-MAC DNA methylation gains occur concomitantly with a loss of DNase I hypersensitivity and activating histone marks, and SIRT1/2 inhibition upregulates activating histone marks. (**A**) DNase-seq and ChIP-seq data of H3K27ac, H3K4me1 and H3K4me3 of MOs and MACs were downloaded from the Blueprint database (refer to Materials and Methods for details). Odds ratios were calculated for bins of 10 bp up to ±2500 bp centering around hypo- (left panel) and hypermethylated (right panel) CpGs in which CpGs annotated in the EPIC 850K array were used as background. (**B**) Schematic representation of DNase I hypersensitivity, H3K27ac, H3K4me3 and H3K4me1 marks near hypermethylated CpGs in *ADORA2A*, *RUNX3*, *IL2RA* and *JAK3*. MOs are represented in red and MACs are represented in blue. (**C**) Chromatin immunoprecipitation (ChIP) of H4K16ac (yellow), H3K4me1 (green), H3K4me3 (light blue) and H3K27ac(dark blue) MOs, MACs and LPS-activated MACs in the presence and absence of 50 μM cambinol. ChIP amplicon positions in relation to hypermethylated CpGs and genes *ADORA2A*, *RUNX3*, *IL2RA* and *JAK3* are depicted in (**B**). Three independent experiments were performed and statistical significance was calculated using paired Student's *t*-tests (*P-value < 0.05, **P-value < 0.01 and ***P-value < 0.001).

Our results showed that the activities of SIRT1/2 determined *de novo* DNA methylation during MO-to-MAC differentiation. It is likely that acquisition of DNA methylation is accompanied by additional histone modifications. Hence, we analyzed the enrichment of histone marks H3K9/14ac, H3K4me3, H3K4me1, H3K36me3, H3K27me3 and H3K27ac, as well as DNase I hypersensitive sites, all obtained from the Blueprint database, in MOs and MACs within a window of 4000 bp centring around hyper- and hypomethylated CpGs (Figure 4A). We observed that for CpGs whose hypermethylation during MO-to-MAC differentiation was blocked in cambinol-treated MACs, they appeared to undergo a dramatic decrease in DNase I hypersensitivity and reduced H3K4me3 and H3K27ac histone marks, which were in accordance to a more closed chromatin profile (39,40). We also observed a slightly higher enrichment of H3K4me1 in MACs with respect to MOs, which marks primed enhancers (41). Conversely, we observed that for CpGs whose MO-to-MAC demethylation was not affected by SIRT1/2 inhibition, they underwent an increase in DNase I accessibility, concurrently with higher enrichment in H3K4me3, H3K27ac and H3K4me1 in MACs compared to MOs. No significant differences were observed for H3K27me3, H3K36me3 and H3K9/14ac, which was due, in part, to lack of data to generate sufficient and reliable reads (Supplementary Figure S3A). Altogether, these results suggest that gains and losses of DNA methylation during MO-to-MAC differentiation occur concomitantly with changing histone marks and chromatin accessibility, in which SIRT1/2 inhibition specifically inhibited MO-to-MAC gains of methylation. Hence, it is not unbefitting to envision that SIRT1/2 may have additional implications in the modification of the chromatin landscape.

To further understand the hierarchy between the establishment of histone marks by SIRT1/2 and DNA methylation and their effect on gene expression, we performed ChIP analyses of H3K4me1, H3K4me3, H3K27ac and H4K16ac in MOs, MACs and cambinol-treated MACs, both before and after LPS activation, in LPS-responsive genes with hypermethylated CpGs. First, we observed a general loss of active histone marks in the MO-to-MAC differentiation process, which was in accordance with what was observed in the Blueprint data (Figure 4B). Second, we observed that SIRT1/2 inhibition by cambinol not only resulted in enrichment of H3K16ac but also in H3K4me3 and H3K27Ac at several proinflammatory loci, both before and following LPS challenge (Figure 4C), and these included *ADORA2A*, *RUNX3* and *JAK3*. A similar trend was observed in *IL2RA*, however it did not reach statistical significance. These results indicated that SIRT1/2 are required to maintain a closed chromatin structure at proinflammatory loci through DNA hypermethylation and the inhibition of activating histone marks. Interestingly, cambinol treatment already results in histone modifications prior to MAC activation, however, aberrant upregulation of these genes was mostly observed following LPS stimulation (Figure 3C). These results suggest that alteration of the histone modification status was not sufficient to induce aberrant expression, hence an additional stimulus was required. In all, our results proved that SIRT1/2 are essential in the establishment of chromatin structure at pro-inflammatory genes, as their functions are not only critical to achieve correct gains of DNA methylation during MO-to-MAC differentiation but is also required for both the loss of active histone marks and their maintenance during differentiation and activation respectively.

### Inhibition of DNA methylation alone is sufficient to aberrantly upregulate proinflammatory genes in LPS-activated MACs

**Figure 5.**
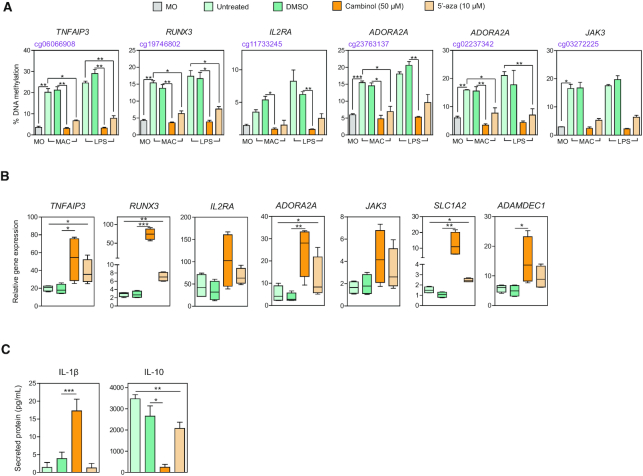
Inhibition of DNA methylation alone is sufficient to upregulate gene expression following LPS exposure. (**A**) Pyrosequencing of hypermethylated CpGs mapped to *TNFAIP3*, *RUNX3, IL2RA, ADORA2A*, and *JAK3* in MOs (gray), MACs and LPS-activated MACs in the presence of DMSO (green), 50 μM cambinol (orange) and 10 μM DAC (light orange) or remained untreated (light green). Untreated MACs were controls for DAC and DMSO-treated MACs were controls for cambinol. Three independent experiments were performed. (**B**) Gene expression analysis of *TNFAIP3*, *RUNX3*, *IL2RA*, *ADORA2A*, *JAK3*, *SLC1A2*, and *ADAMDEC1* of MACs differentiated in the presence of 50 μM of cambinol or 10 μM of DAC and subsequently activated with 10 ng/mL of LPS for 18 hrs. Gene expression were normalized against housekeeping gene *RPL38* and represented as fold changes against control (MACs treated with corresponding volumes of DMSO for cambinol or untreated for DAC). Four independent experiments were performed. (**C**) ELISA analyses of IL-1β and IL-10 secretion in M-CSF MACs differentiated for 5 days and activated with 10 ng/mL LPS for an additional 18 hrs in the presence of 50 μM of cambinol or 10 μM of DAC. DMSO-treated and untreated MACs were used as controls. Four independent experiments were performed. All statistical analyses were performed using paired Student's *t*-tests (*P-value < 0.05, **P-value < 0.01 and ***P-value < 0.001).

The strict association between the cambinol-mediated aberrant upregulation of genes following encounter with LPS stimulus and their previous inhibition of DNA methylation led us to speculate on the direct relevance of DNA methylation in the transcriptional status of pro-inflammatory genes following activation. To test such hypothesis, we differentiated MACs in the presence of a bona fide DNMT inhibitor, 5-aza-2′-deoxycytidine (DAC) (42). Firstly, utilizing pyrosequencing, we confirmed that MACs differentiated in the presence of cambinol and DAC displayed an effective inhibition in gains of DNA methylation both before and after LPS exposure (Figure 5A). Secondly, in parallel to what was observed in cambinol-treated cells, we also observed a significant upregulation of such LPS-induced genes as *ADORA2A*, *RUNX3*, *TNFAIP3* and *SLC1A2* in MACs treated with DAC following LPS stimulation (Figure 5B). Other genes such as *IL2RA, JAK3* and *ADAMDEC1* displayed a trend in their upregulation without reaching statistical significance (Figure 5B). Interestingly, we did not observe an upregulation in these genes prior to LPS stimulation (Supplementary Figure S4A), suggesting that, similar to cambinol, LPS stimulus was required for their aberrant expression. Finally, DAC-treated MACs secreted significantly less IL-10 following LPS stimulus (Figure 5C), however no changes in IL-1β secretion were observed. Altogether, these results suggested that the inhibition of DNA methylation alone by DAC is sufficient to induce transcriptional activation of proinflammatory genes in the presence of functional sirtuins, and, to some extent, mediated a change in the final functional phenotype of MACs.

### Both SIRT1 and SIRT2 interact with DNMT3B and are recruited to genes that become hypermethylated during macrophage differentiation

**Figure 6.**
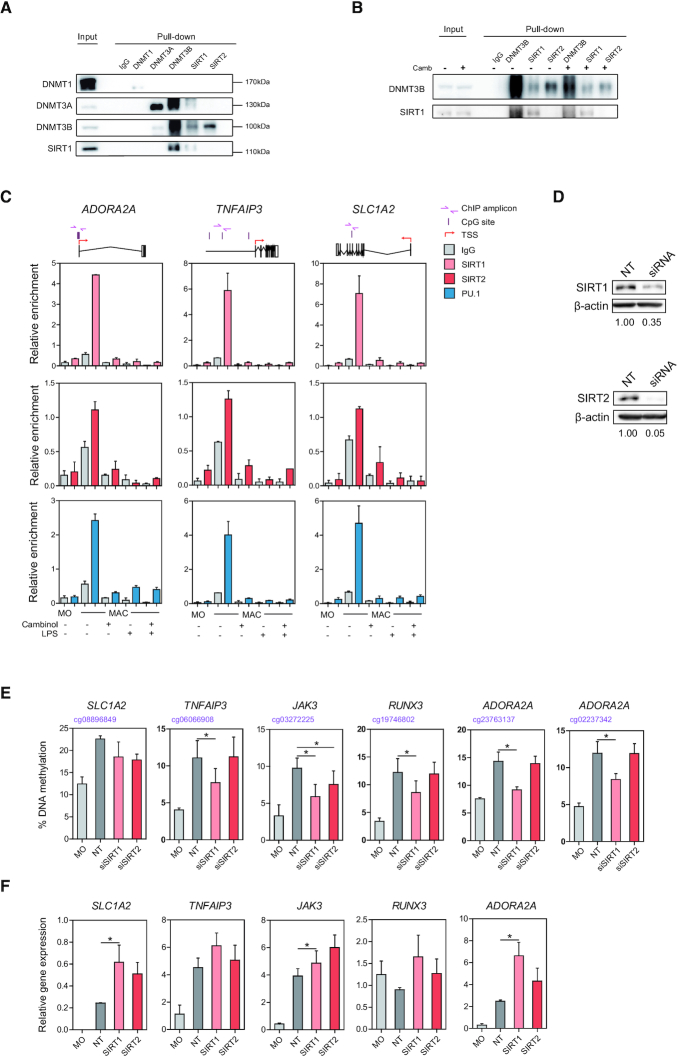
SIRT1 and 2 interact with DNMTs to drive DNA hypermethylation at inflammatory loci. (A) Co-immunoprecipitation assays were performed in MOs differentiated to MACs in the presence of M-CSF for 5 days. Protein extracts were immunoprecipitated utilising anti-DNMT1, -DNMT3A, -DNMT3B, -SIRT1 and –SIRT2 antibodies, in which IgG was used as a negative control and total protein extract was used as input. (B) Co-immunoprecipitation of MACs differentiated in the presence of DMSO or 50 µM cambinol for 5 days. (C) Chromatin immunoprecipitation of two independent experiments of SIRT1 (pink), SIRT2 (red) and PU.1 (blue) was performed in isolated monocytes and MACs differentiated in the presence of M-CSF for 5 days in the presence and absence of cambinol, before and after LPS stimulation for 18 hrs. (D) MOs were transfected with 100nM of corresponding siRNA and differentiated to MACs in the presence of M-CSF for 5 days, as described in Materials and Methods. Annotated numbers indicate relative band intensities compared to non-targeting control (NT). (E) Pyrosequencing of hypermethylated CpGs in MOs and MACs transfected with NT, siSIRT1 and siSIRT2 prior to LPS activation. (F) Gene expression of *SCL1A2*, *TNFAIP3*, *JAK3*, *RUNX3* and *ADORA2A* in MOs and LPS-activated MACs transfected with NT, siSIRT1 and siSIRT2, as normalised against *RPL38*. (D-F) Statistical significance of at least three independent experiments was calculated using paired student t-tests (* p-value < 0.05, ** p-value < 0.01 and *** p-value < 0.001).

To better understand the relationship between SIRT1/2 and the DNA methylation machinery in MACs in relation to the control of proinflammatory genes, we then performed co-immunoprecipitation experiments to assess the potential interaction between SIRT1/2 with DNMTs. Immunoprecipitation, performed using M-CSF MACs differentiated from MOs for 5 days, showed that both SIRT1 and SIRT2 co-immunoprecipitated with DNMT3B, but not DNMT1 and DNMT3A (Figure 6A). Interestingly, DNMT3B and DNMT3A also seemed to interact with each other (Figure 6A). Such interaction has been previously described in other cell types (43,44). Furthermore, the interaction between DNMT3B and SIRT1/2 was not affected by cambinol treatment (Figure 6B). Subsequently, we tested the recruitment of sirtuins to inflammatory loci that become hypermethylated during MAC differentiation in the presence of cambinol in unstimulated and LPS-activated MACs. We first observed an increase in SIRT1 and SIRT2 binding to *SLC1A2*, *TNFAIP3* and *ADORA2A* loci upon MO-to-MAC differentiation, in which the complex appeared to dissociate following LPS stimulation. Second, we observed that their binding was abrogated upon inhibition with cambinol both before and after LPS stimulation (Figure 6C). Finally, and remarkably, we also observed binding of PU.1 to these genes, in which the pattern of binding of PU.1 was very similar to SIRT1 and 2 (Figure 6C). We were unable to detect binding of DNMT3A/B to these sequences, however, we could not discard that this was a technical limitation due to the quality of the DNMT antibodies.

Utilizing specific pharmacological inhibitors targeting SIRT1 and SIRT2, we observed that inhibition of SIRT1 may have a stronger effect on gene expression than SIRT2 (Supplementary Figure S1C). Thus in order to assess the specific contribution of sirtuins to DNA hypermethylation and expression of inflammatory genes, we downregulated SIRT1 and SIRT2 using siRNAs. We were able to downregulate SIRT1 and SIRT2 with distinct efficiency, achieving 65% and 95% reduction in protein expression respectively (Figure 6D). We observed that upon SIRT1 knockdown, there was significant inhibition in the acquisition of MO-to-MAC DNA methylation for CpGs annotated to *TNFAIP3*, *JAK3*, *RUNX3* and *ADORA2A*. Conversely, SIRT2 knockdown did not appear to have an effect on DNA methylation, except in the case of *JAK3* (Figure 6E). Knockdown of either sirtuins did not have an effect on hypomethylated CpGs (Supplementary Figure S4A). Moreover, the inhibition of DNA methylation gain observed in *ΔSIRT1* MACs correlated with an aberrant upregulation of the same genes following LPS stimulation, albeit some did not reach statistical significance (Figure 6F). Similar to what was observed with pharmacological inhibitors, SIRT1 downregulation appeared to have stronger impact compared to SIRT2. These observations suggest that SIRT1 is the main player mediating the acquisition of DNA methylation during MAC differentiation.

## DISCUSSION

In this study, we report a novel role for SIRT1/2 in macrophage differentiation, in which these deacetylases act as a key element in establishing the hierarchy of epigenetic modifications involving acquisition of *de novo* DNA methylation through direct DNMT3B interaction, linked to H3K4me3 demethylation and H3K27ac deacetylation, to prevent premature activation of pro-inflammatory genes during MAC differentiation and prior to encounter with bacterial antigens. 

Our initial observation showed that both SIRT1 and SIRT2 undergo a drastic upregulation concomitant with MAC differentiation, and that the effects of SIRT1/2 inhibition during this process impact the expression levels of many genes related to the immune properties of these cells, with a particular effect on proinflammatory genes mediating their aberrant upregulation. These observations support a major role of SIRT1/2 in maintaining a repressed status for these genes, most likely through the direction interaction with DNMTs, which is in concordance to what has been observed in other studies. Notably, SIRT1 has been previously described by Park *et al.* to exert anti-inflammatory effects in RA macrophages through the inhibition of NF-κB signalling, and SIRT1 Tg-mice displayed reduced M1 polarization (10). Individual siRNA knockdown of SIRT1 and SIRT2 showed that the former mediated loss of DNA methylation and upregulated proinflammatory gene expression while the latter had weaker significant effects. It is therefore likely that SIRT1 is the major contributor to the phenotype of M-CSF macrophages while SIRT2 may offer little or no contribution.

Interestingly, the analysis of DNA methylation changes in relation to inhibition and downregulation of SIRT1/2 shows that only gains of DNA methylation are affected, in contrast to DNA demethylation, which is identical to those without SIRT1/2 inhibition. Remarkably, the group of genes that gain methylation during macrophage differentiation, almost all of which are affected by SIRT1/2 inhibition, associate with additional epigenetic features including a decrease in H3K27ac and H3K4me3 and an increase in H3K4me1. These observations indicate that these three histone marks are linked to the observed gains of DNA methylation. Different studies have established links between DNA methylation and histone modifications. First, the presence of DNA methylation has originally been described to prevent methylation of H3K4 (45). Second, treatment with DNMT inhibitors were shown to increase H3K4me2 and H3K4me3 occupancy at target promoters and gene bodies, as well as resulting in a dramatic increase in enhancer activity (46). Furthermore, structural and biochemical studies have shown a direct interaction between the catalytic domain of *de novo* DNA methyltransferases with unmethylated H3K4 histone peptide leading to the catalytic activation of DNMT3A (47,48). This interaction was in turn inhibited by H3K4 methylation and histone acetylation (49). Taken together, evidence suggests an inverse relationship between DNA methylation and H3K4 methylation. Third, histone remodelers of H3K4 have been linked to DNMT3 recruitment and activity, in which MLL4 enhances DNMT3A-catalyzed DNA methylation at super-enhancers (50), and contradictorily LSD1 has been described to function in a complex with DNMT3 to mediate both H3K4 demethylation and DNA methylation at pluripotentency enhancers (51). Finally, DNA methylation has been described to inversely correlate with acetylated H3K27 deposition, a classical marker for enhancers (39), however, recent studies also identified bivalent regions, in which H3K27ac co-exists with DNA methylation, although this accounts for a minority of CpGs (38%) (52). Our findings reinforce the connection between DNA methylation and H3 modifications, in which DNA methylation levels inversely correlate with H3K4me3 and H3K27ac (Figure 4), as such that the inhibition of SIRT1/2 mediates higher enrichment of these two histone marks concurrently with a decrease in DNA methylation prior to encounter with bacterial antigen. Furthermore, CpGs that become demethylated upon SIRT1/2 inhibition also gain H4K16ac, which is coherent with the role of SIRT1/2 in its deacetylation. Altogether, these results indicate that the activities of SIRT1/2 are essential in the maintenance of a silent chromatin, defined by DNA hypermethylation and low levels of H3K4me3, H3K27ac and H4K16ac, and their inhibition results in chromatin aperture.

The dynamics between SIRT1/2 and DNMTs has been partly elucidated by our results, in which we have observed a direct interaction between SIRT1/2 and DNMT3B, which in turn interacts with DNMT3A. Other studies had previously linked SIRT1 activity with DNMTs. For instance, O’Hagan *et al.* showed that SIRT1 recruitment to induced double-strand breaks was dependent on DNMT1, and that DNMT1, DNMT3B and SIRT1 form part of a large multi-protein complex following oxidative DNA damage (43). We observe that the binding of SIRT/DNMT3B complex to chromatin appears to be dependent on SIRT1/2 activity, and it is possible that other transcription factors, such as PU.1, may target the complex to repress pro-inflammatory genes in M0 MACs. Furthermore, the interaction of SIRT1/2 with DNMTs may also participate in the direct deacetylation of these enzymes to modify their activities in a similar way that has been observed for SIRT1 and DNMT3L (53). Additionally, SIRT1 has also been indirectly linked to DNA methylation through its activities of modifying histones. One such example was described by Aguilar-Arnal *et al.* where SIRT1 was observed to directly deacetylate MLL1 and modulate H3K4 tri-methylation along the circadian cycle (54). Since H3K4 methylation play concerted roles with DNA methylation, it is therefore plausible that SIRT1 can modulate DNA methylation through histone modifications.

The hierarchy of DNA methylation, histone modifications and gene expression has been proven to be extremely complex, in which contradictory literature has described DNA methylation as both a cause and a consequence of changes in gene expression. While it is generally accepted that DNA methylation results in gene repression, however, recent studies have provided conflicting evidence in their relationship. One study involving 62 unrelated individuals concluded that DNA methylation contributes to only 8% of inter-individual gene expression variation (55). Similarly, another study shows that the presence of DNA methylation is insufficient to transcriptionally repress promoters (56). This conclusion was, however, recently refuted by Korthauer *et al.*, as the authors show that, by utilizing different statistical strategies that include statistical inference of differentially methylated regions and additional normalization techniques, promoter DNA methylation indeed correlate negatively with gene expression (57). Nevertheless, the role of DNA methylation during a cell activation event, such as encounter with bacterial antigens, is still unknown. In a study conducted in our laboratory, Vento-Tormo *et al.* show that a previous demethylation step, together with histone modifications, is required for correct upregulation of inflammatory genes during dendritic cell activation (28). Contrastingly, a more recent article shows that upregulation of proinflammatory genes precedes DNA demethylation following dendritic cell activation, however, this behavior only accounts for less than 10% of all differentially expressed genes and differentially methylated CpGs (58). In this study, we provide a direct link between DNA methylation, histone modifications and gene expression. We show that SIRT1/2-dependent establishment of correct chromatin state prior to LPS challenge is essential for proper and controlled expression of proinflammatory genes following macrophage activation. In fact, we demonstrate that the majority of DNA methylation gains and histone modifications are established in the MO-to-MAC differentiation step, similar to what were observed by Vento-Tormo and colleagues, and their disruption by cambinol results in aberrant post-LPS upregulation of proinflammatory genes. Furthermore, we demonstrate the essentiality of DNA methylation in gene expression by specifically inhibiting MO-to-MAC DNA hypermethylation utilizing DAC, and we show that although DAC inhibits methylation prior to LPS challenge, aberrant upregulation of gene expression is not observed until after MAC activation.

In conclusion, our work directly identifies SIRT1/2, through direct interaction with DNMTs, as essential factors in the establishment of gains of methylation, as well as additional epigenetic modifications that prevent premature expression of proinflammatory genes during differentiation to MACs and prior to their encounter to bacterial antigens.

## DATA AVAILABILITY

Expression and methylation array data for this publication have been deposited in the NCBI Gene Expression Omnibus and are accessible through GEO Series accession numbers GSE131115 and GSE131177.

## Supplementary Material

gkz1127_Supplemental_File
