# Supplementary material for: SIRT1/2 orchestrate acquisition of DNA methylation and loss of histone H3 activating marks to prevent premature activation of inflammatory genes in macrophages
Source: Nucleic Acids Res. 2019 Dec 4;48(2):665–81. doi: 10.1093/nar/gkz1127 (PMC6954413; doi:10.1093/nar/gkz1127)
Supplement: gkz1127_Supplemental_File [file gkz1127_supplemental_file.pdf]

Supplementary Figure Legends

Supplementary Figure 1. (A) Western blot analysis of protein expression of SIRT1, acetylated lysine 310 of p65 (p65-AcK310), total H3 in MOs, and M-CSF MACs differentiated for 5 days in the presence of 50 µM of cambinol or equivalent volumes of DMSO. Protein expression of total H3 was visualised as loading control. The right panel bar graph represents the ratio of band intensities of p65-AcK310 and total H3. (B) RT-qPCR analyses of *IL-1A*, *IL-1B*, *TNF* and *CD163* were performed in MOs (gray), MACs (blue) and MACs activated by LPS (orange) in the presence of 70 µM EX-527 for SIRT1 inhibition, 4 µM AGK2 for SIRT2 inhibition, in combination or 50 µM cambinol. Treatment with DMSO was used as control. Relative RNA expression was calculated by normalising against housekeeping gene *RPL38*. Statistical significance was calculated using ΔCt values by paired student t-test (* p-value < 0.05, ** p-value < 0.01 and *** p-value < 0.001). (C) GSEA analyses of cambinol-upregulated (left panel) and -downregulated (right panel) genes obtained from expression microarray. Ranking using log2FC of gene expression of cambinol-treated MACs in respect to control MACs was performed and Normalized Enrichment Scores (NES) were calculated for the relevant hallmarks. Positive and negative NES indicate positive and negative enrichment, respectively, of the specific hallmarks.

Supplementary Figure 2. (A) Distribution of CpGs in relation to CpG islands (left panel) and in relation to gene (right panel) according to annotations obtained from Infinium MethylationEPIC array. Proportion of each category was calculated for background, and hypo- and hypermethylated CpGs and represented as a percentage. Abbreviations: N - north; S - south; 1stExon - first exon; UTR - untranslated region; ExonBnd - exon boundary; TSS - transcription start site. (B) Gene ontology analysis of hypomethylated CpGs mapped to genesusing GREAT online tool (http://great.stanford.edu/public/html) by applying the basal plus extension settings. CpGs annotated in the EPIC 850K array were used as background. (C) HOMER TF motif enrichment analysis of hypomethylated CpGs were carried out using a window of ±250 bp centring around the CpGs. CpGs annotated in the EPIC 850K array were used as background. (D) Representation of the beta values

of selected hypomethylated CpGs that map to relevant genes *TM7SF4*, *ACP5* and *ADAM12*. (E) Western blot analyses of DNMT1, DNMT3A and DNMT3B protein expression in MO, MACs and LPS-activated, in the presence of DMSO or cambinol. Protein expression of β-Actin was used as loading control. Relative protein expression was calculated by normalising band intensities of each protein against β-Actin. Bar graphs represent the mean and standard deviation of relative protein amounts from four independent experiments. A representative western blot is displayed.

Supplementary Figure 3. (A) ChIP-seq data of H3K27me3, H3K36me3 and H3K9/14ac of MOs and MACs were downloaded from the Blueprint database (refer to Materials and Methods for details). Odds ratios were calculated for bins of 10 bp up to ±2500 bp centering around hyper- (left panel) and hypomethylated (right panel) CpGs, in which CpGs annotated in the EPIC 850K array were used as background.

Supplementary Figure 4. (A) Gene expression analysis of *IL2RA*, *ADORA2A*, *SLC1A2*, and *ADAMDEC1* of MACs differentiated in the presence of 10 µM of DAC (5'-aza) prior to LPS activation. Gene expression were normalised against housekeeping gene *RPL38*. (B) Pyrosequencing of hypomethylated CpGs cg25431432, cg23898184 and cg13075942 annotated to *HIF1A*, *UCHL5* and *RAD51L1*, respectively, in MO and MACs transfected with non-targeting siRNA and siRNA targeting SIRT1 and SIRT2 in the absence of LPS stimulation.

Supplementary Figure 1

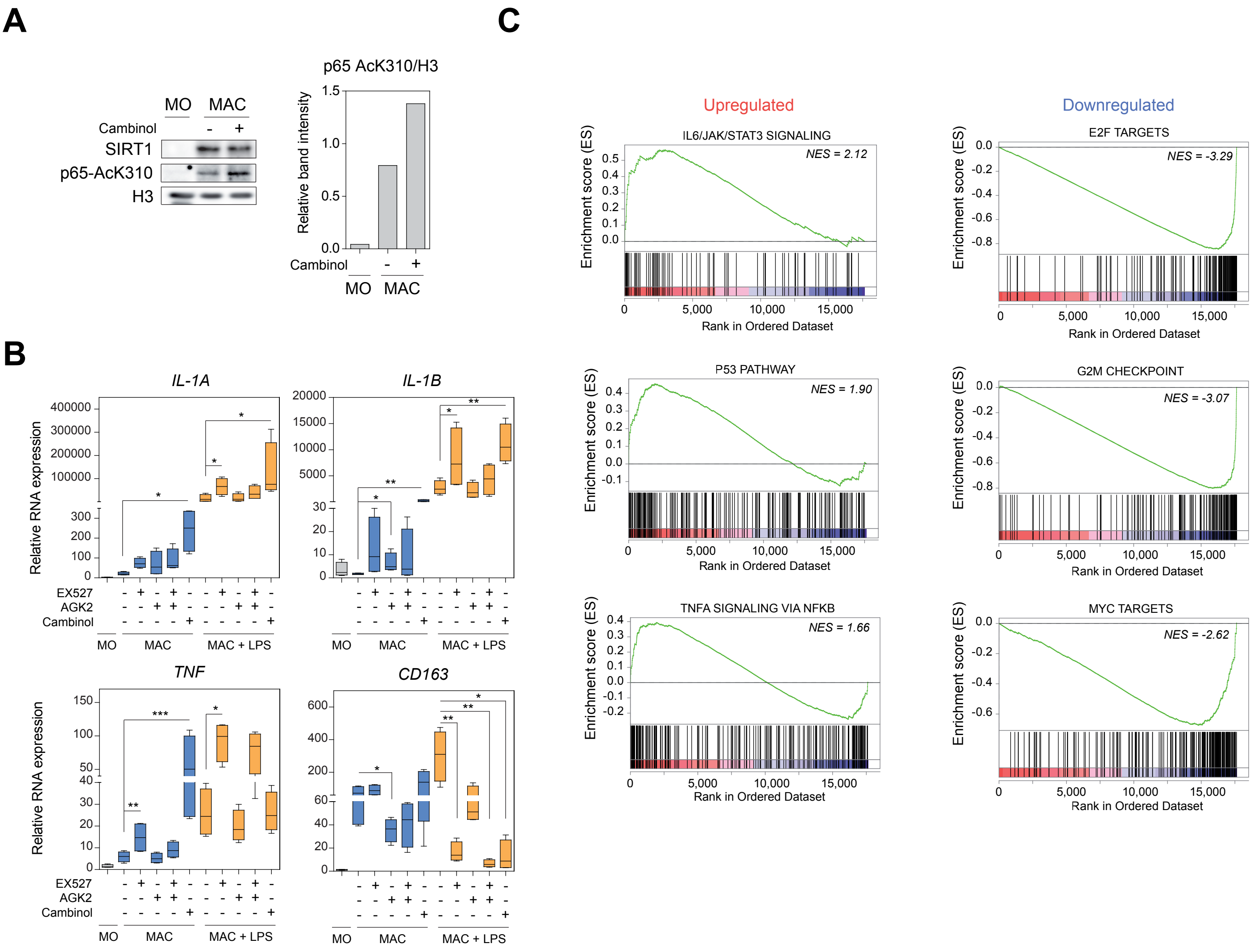

Supplementary Figure 2

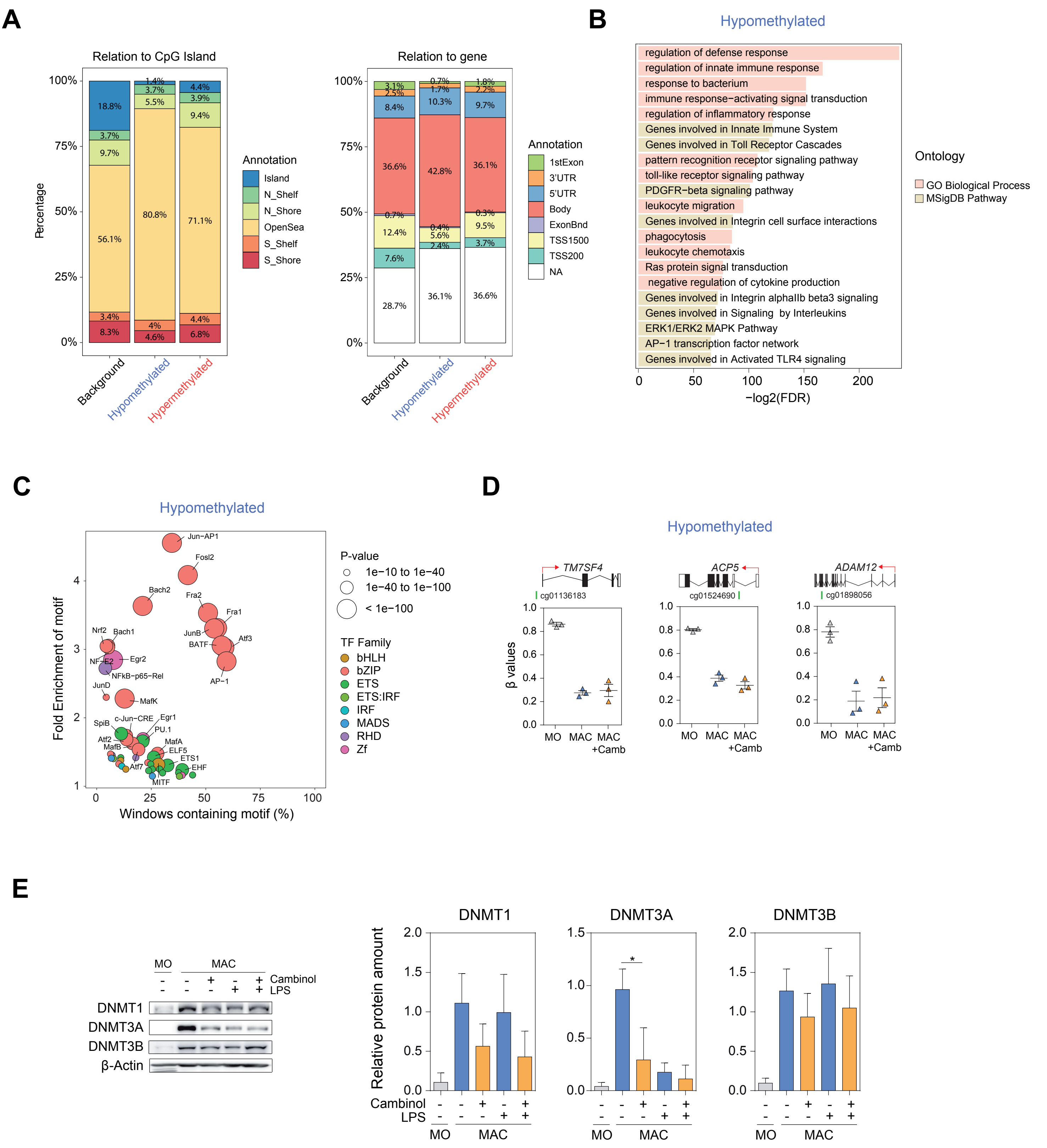

Supplementary Figure 3

A

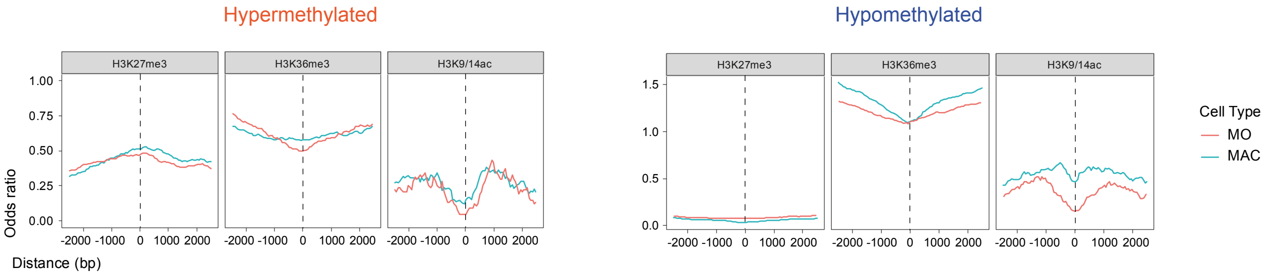

Supplementary Figure 4

A

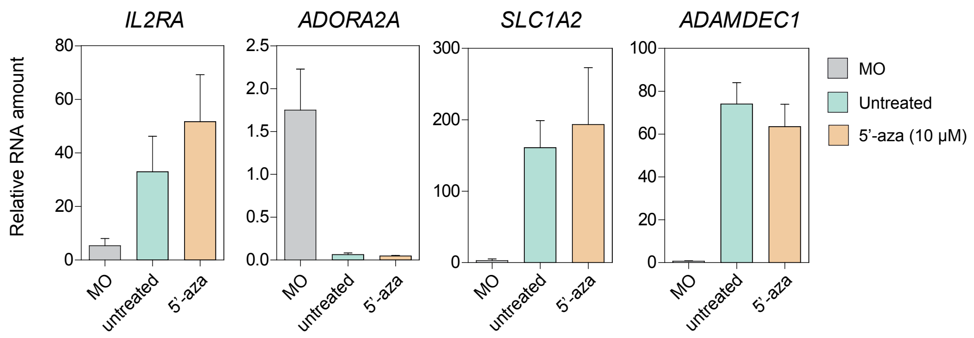

B

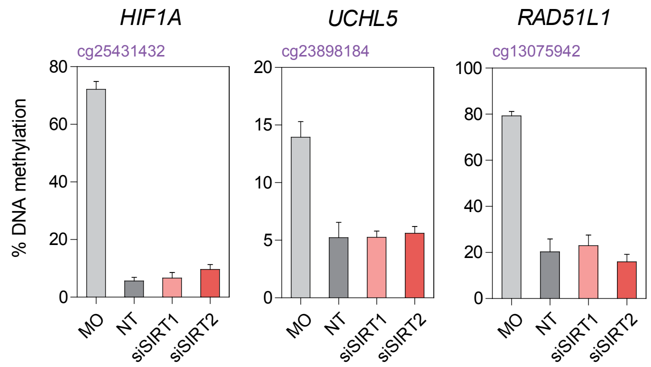

Supplementary Table 1. List of primers used in this study

**RT-qPCR Primers**			
Gene	Primer ID	Primer type	Primer sequence
RUNX1	RT_Hsa_RUNX1_F	Forward	GCAGGATACAAGGCAGATCC
	RT_Hsa_RUNX1_Rv	Reverse	ACAGAAGGAGAGGCAATGG
TNFAIP3	Hs_TNFAIP3_Primer_F	Forward	CCATGGCACAACTCATCTCA
	Hs_TNFAIP3_Primer_R	Reverse	GCCATTTCTTGTACTCATGC
JUN	JUN_RT_F1	Forward	TGGGCTATTTTTAGGGGTTG
	JUN_RT_R1	Reverse	TGGGCAGTTAGAGAGAAGGTG
NR4A3	NR4A3_RT_F1	Forward	GTCCGCTCCTCCTACACTCT
	NR4A3_RT_R1	Reverse	CGCATAACTGGAACCTGGA
RUNX3	RUNX3_RT_F1	Forward	ACTCAGCACCACAAGCCACT
	RUNX3_RT_R1	Reverse	GGAAGGAGCGGTCAAACTG
ADAMDEC1	ADAMDEC1_RT_F1	Forward	AACAATCGACGTGTGGGACT
	ADAMDEC1_RT_R1	Reverse	TGGAACATCAGGCATACCAA
SLC1A2	SLC1A2_RT_F1	Forward	CAAATGAATGGTGTTGTCCTG
	SLC1A2_RT_R1	Reverse	AGGCTGATGTCCTCTGTTGG
IL2RA	IL2RA_RT_F1	Forward	GTCAGCAACATGCTCCAGAAG
	IL2RA_RT_R1	Reverse	AGGCAACTCTCATTCTTGGTTA
INHBA	Hs_INHBA_qPCR_F	Forward	AGTGCCAATACCATGAAGAGGA
	Hs_INHBA_qPCR_R	Reverse	AGCAAATTCTCTTTCTGGTCCC
CD38	CD38_RT_F1	Forward	ATCCTGGTCCTGATCCTCGT
	CD38_RT_R1	Reverse	AATGCACCCTTGAAAGCATC
TNFAIP8	TNFAIP8_RT_F1	Forward	CCGAAGCAGAAGAATCCAAG
	TNFAIP8_RT_R1	Reverse	GCTCATCCAGCACCTCACTAC
ADORA2A	ADORA2A_RT_F2	Forward	CTCTTCATTGCCTGCTTCGT
	ADORA2A_RT_R2	Reverse	AGTGGTTCTTGCCCTCCTTT
CD83	CD83_RT_F1	Forward	GGATGGGCAGAGAAACCTAA
	CD83_RT_R1	Reverse	AGAAAATAACCAGAGCCAGCA
JAK3	JAK3_RT_F2	Forward	CTCCTTCCGAGCCGTCATT
	JAK3_RT_R2	Reverse	ACCATTCCACAGCCCATC
**ChIP Primers**			
Gene	Primer ID	Primer type	Primer sequence
RUNX3	RUNX3_ChIP_F1	Forward	AAAGCCCCATTCTCCTGGT
	RUNX3_ChIP_R1	Reverse	CCCTCGCAACAGGTTCTTC
IL2RA	IL2RA_ChIP_F2	Forward	GTCACCCTTGTGGGTCCAT
	IL2RA_ChIP_R2	Reverse	CTAGGCAGTTTCCTGGCTGA
ADORA2A	ADORA2A_ChIP_F2	Forward	AGGTGCCAATCCTTCACG
	ADORA2A_ChIP_R2	Reverse	TCCATGTCACAGTCCCTGAG
JAK3	JAK3_ChIP_F1	Forward	CCGGTAAGCGATCAACAAAC
	JAK3_ChIP_F2	Reverse	TTCCCACAGTAACCACCACA
TNFAIP3	TNFAIP3_ChIP_F3	Forward	TCTGGGAGTTGACTTGACAGC
	TNFAIP3_ChIP_R3	Reverse	CCAATCTTCAGGCCACATTT
SLC1A2	SLC1A2_ChIP_F1	Forward	TTCCTCTTACCCCACCCTTT
	SLC1A2_ChIP_R1	Reverse	GCCACCAACCACAACTGA
**Pyrosequencing Primers**			
Gene (CpG ID)	Primer ID	Primer type	Primer sequence
TNFAIP3 (cg06066908)	TNFAIP3_PS_F2	Forward	TTGAGAAGGATTTAGAGGTGTTAT
	TNFAIP3_PS_R2_Btn	Reverse	CAAACCACATTTCTACTATACTATCTTAAC[Btn]
	TNFAIP3_PS_Seq2	Sequencing	TGAAGGAGGTTGATAGA
RUNX3 (cg19746802)	RUNX3_PS_F1_Btn	Forward	[Btn]GAATTTGTTGAGAGGGGAGAGG
	RUNX3_PS_R1	Reverse	TATCAACAAACATTTCCTAAATACCC
	RUNX3_PS_Seq1	Sequencing	ACCCACTACCAATAAAAAACTATAC
ADORA2A (cg23763137, cg02237342)	Hs_PS_ADORA2A_F	Forward	TGTTAGTTATTTGAGGGAGGTGTAGGT
	Hs_PS_ADORA2A_R	Reverse	[Btn]CTCCCCACACCCCCATATATC
	Hs_PS_ADORA2A_Seq	Sequencing	GAGGTGTAGGTGTTAAT
IL2RA (cg11733245)	Hs_PS_IL2RA_F	Forward	AGGATGTGGGATGGGAAGAT
	Hs_PS_IL2RA_R	Reverse	[Btn]ACCCACACCTCCAACAATT
	Hs_PS_IL2RA_Seq	Sequencing	TTTTTAAGTATTGGGTTGG
JAK3 (cg03272225)	JAK3_PS_F1	Forward	GGTGGTTTGTTGAGAGTAATTTAGGTTTAA
	JAK3_PS_R1_Btn	Reverse	CAAAACTTCCCTTCCCACAATAACCA[Btn]
	JAK3_PS_Seq1	Sequencing	AATTTGTATATTTTTTGATGATGT
SLC1A2 (cg08896849)	SLC1A2_PS_F1_Btn	Forward	[Btn]TTTTGGTTTGGGGTAGGAAGAA
	SLC1A2_PS_R1	Reverse	TAACCACCAACCACAACTAAACAATC
	SLC1A2_PS_Seq1	Sequencing	CAATCCTAATATCCTAACAAC

HIF1A (cg25431432)	HIF1A_PS_F1	Forward	TTTTTGAATGAATAGAGTAGTTAGGAATTT
	HIF1A _PS_R1_Btn	Reverse	AAAATTTAAACCCTAAAACTAAATCTACTC[Btn]
	HIF1A _PS_Seq	Sequencing	ATATTATGTTATTAGATTTTTATG
UCHL5 (cg23898184)	UCHL5_PS_F1_Btn	Forward	[Btn]ATTTGGGTTATAAAGTTAGTTTAAGTTGA
	UCHL5_PS_R1	Reverse	CCACAATTTCAAAAAACTACACTAAAC
	UCHL5_PS_Seq	Sequencing	ATATAAACTATAAATCTTCCC
RAD51L1 (cg13075942)	RAD51L1_PS_F1_Btn	Forward	[Btn]GGGTTTTGGGGTTTGGTTGGATAAAATA
	RAD51L1_PS_R1	Reverse	CTCCTAACCCCTATAAAAACTACTTCT
	RAD51L1_PS_Seq	Sequencing	CTCCCTACAAAAACTTTC

Supplementary Table 2. List of inflammation-relevant genes overexpressed in cambinol-treated M-CSF macrophages

Gene	Log_2FC	FDR	Relevance in inflammatory response
FABP4	2.90	0.000425	Intracellular lipid chaperone and adipokine required for neutrophil recruitment and bacterial clearance (1)
PDK4	2.06	0.000619	Mitochondrial metabolic regulator with a role in classical proinflammatory activation of macrophages (2)
IL1B	1.94	0.000701	A potent proinflammatory cytokine (3)
SLC1A2	1.65	0.013180	This gene encodes a member of a family of solute transporter proteins that becomes upregulated in activated macrophages (4)
CXCL8	1.59	0.003263	Encodes the protein IL-8 which is an important inflammatory chemokine produced by macrophages to recruit neutrophils (5)
PDPN	1.57	0.003263	Transmembrane receptor glycoprotein that is upregulated in inflammatory macrophages (6)
MMP2	1.45	0.000873	A member of the matrix metalloproteinase (MMP) gene family that promotes chemokine-induced leukocyte migration (7)
DUSP1	1.39	0.000551	Induced by proinflammatory stimuli, which dephosphorylates and inactivates MAPKs to enforce the off-phase of the inflammatory response (8)
IL7R	1.37	0.038196	Receptor for IL-7, in which both proteins have been implicated in rheumatoid arthritis pathogenesis (9)
IL1R2	1.30	0.019093	Acts as a decoy receptor to attenuate IL-1 activity, thus turning off the proinflammatory response (10)
CXCL5	1.25	0.000987	Proinflammatory chemokine associated with neutrophil recruitment and monocyte migration (11)
CMKLR1	1.18	0.000953	G protein-coupled receptor (GPCR) implicated in macrophage-mediated inflammation by promoting macrophage adhesion (12)
MME	1.14	0.002298	Proteinase required for macrophage-mediated extracellular matrix proteolysis and tissue invasion, processed associated with inflammatory processes (13)
ALOX15B	1.14	0.002251	Enzyme that oxidizes fatty acids to substances to promote local inflammation (14)
CIITA	1.13	0.001783	Transcription coactivator that controls the expression of MHC class II genes (15)
CD86	1.13	0.000641	Receptor involved in the co-stimulatory signal essential for T cell proliferation and IL-2 production (16)
S100A8	1.12	0.013650	Activates NF-kB pathway in macrophages and promoted the expression of inflammatory cytokines in the microenvironment (17)
CCL18	1.08	0.011160	Secreted by M2 macrophages and is involved in the migration, invasion and epithelial-to-mesenchymal transition of cancer cells (18)
ADAMDEC1	1.06	0.002115	A metallopeptidase that is induced in macrophages in response to bacterial antigens (19)

SMAD3	1.00	0.004980	Major component of TGF-β pathway involved in inflammation (20)
TNFAIP3	0.77	0.003072	Required for LPS-induced production of proinflammatory cytokines and IFN-β in LPS-tolerised macrophages (21)
RUNX3	0.72	0.015218	RUNX3 mediates TGF-β signaling in the monocytic lineage (22)
JUN	0.62	0.037287	Key modulator of macrophage phenotype (23)

Supplementary References

1. Liang,X., Gupta,K., Quintero,J.R., Cernadas,M., Kobzik,L., Christou,H., Pier,G.B., Owen,C.A. and Çataltepe,S. (2019) Macrophage FABP4 is required for neutrophil recruitment and bacterial clearance in Pseudomonas aeruginosa pneumonia. *FASEB J.*, **33**, 3562–3574.

2. Jha,M.K., Song,G.J., Lee,M.G., Jeoung,N.H., Go,Y., Harris,R.A., Park,D.H., Kook,H., Lee,I.-K. and Suk,K. (2015) Metabolic Connection of Inflammatory Pain: Pivotal Role of a Pyruvate Dehydrogenase Kinase-Pyruvate Dehydrogenase-Lactic Acid Axis. *J. Neurosci.*, **35**, 14353–69.

3. Wynn,T.A. and Vannella,K.M. (2016) Macrophages in Tissue Repair, Regeneration, and Fibrosis. *Immunity*, **44**, 450–462.

4. Martinez,F.O., Gordon,S., Locati,M. and Mantovani,A. (2006) Transcriptional profiling of the human monocyte-to-macrophage differentiation and polarization: new molecules and patterns of gene expression. *J. Immunol.*, **177**, 7303–11.

5. Arango Duque,G. and Descoteaux,A. (2014) Macrophage cytokines: involvement in immunity and infectious diseases. *Front. Immunol.*, **5**, 491.

6. Astarita,J.L., Acton,S.E. and Turley,S.J. (2012) Podoplanin: emerging functions in development, the immune system, and cancer. *Front. Immunol.*, **3**, 283.

7. Song,J., Wu,C., Korpos,E., Zhang,X., Agrawal,S.M., Wang,Y., Faber,C., Schäfers,M., Körner,H., Opdenakker,G., *et al.* (2015) Focal MMP-2 and MMP-9 activity at the blood-brain barrier promotes chemokine-induced leukocyte migration. *Cell Rep.*, **10**, 1040–54.

8. Smallie,T., Ross,E.A., Ammit,A.J., Cunliffe,H.E., Tang,T., Rosner,D.R., Ridley,M.L., Buckley,C.D., Saklatvala,J., Dean,J.L., *et al.* (2015) Dual-Specificity Phosphatase 1 and Tristetraprolin Cooperate To Regulate Macrophage Responses to Lipopolysaccharide. *J. Immunol.*, **195**, 277–88.

9. van Roon,J.A.G., Hartgring,S.A.Y., Wenting-van Wijk,M., Jacobs,K.M.G., Tak,P.-P., Bijlsma,J.W.J. and Lafeber,F.P.J.G. (2007) Persistence of interleukin 7 activity and levels on tumour necrosis factor alpha blockade in patients with rheumatoid arthritis. *Ann. Rheum. Dis.*, **66**, 664–9.

10. Shimizu,K., Nakajima,A., Sudo,K., Liu,Y., Mizoroki,A., Ikarashi,T., Horai,R., Kakuta,S., Watanabe,T. and Iwakura,Y. (2015) IL-1 Receptor Type 2 Suppresses Collagen-Induced Arthritis by Inhibiting IL-1 Signal on Macrophages. *J. Immunol.*, **194**, 3156–3168.

11. Yoshida,K., Korchynskyi,O., Tak,P.P., Isozaki,T., Ruth,J.H., Campbell,P.L., Baeten,D.L., Gerlag,D.M., Amin,M.A. and Koch,A.E. (2014) Citrullination of epithelial neutrophil-activating peptide 78/CXCL5 results in conversion from a non-monocyte-recruiting chemokine to a monocyte-recruiting

chemokine. *Arthritis Rheumatol. (Hoboken, N.J.)*, **66**, 2716–27.

12. Hart,R. and Greaves,D.R. (2010) Chemerin Contributes to Inflammation by Promoting Macrophage Adhesion to VCAM-1 and Fibronectin through Clustering of VLA-4 and VLA-5. *J. Immunol.*, **185**, 3728–3739.

13. Shipley,J.M., Wesselschmidt,R.L., Kobayashi,D.K., Ley,T.J. and Shapiro,S.D. (1996) Metalloelastase is required for macrophage-mediated proteolysis and matrix invasion in mice. *Proc. Natl. Acad. Sci. U. S. A.*, **93**, 3942–6.

14. Magnusson,L.U., Lundqvist,A., Karlsson,M.N., Skålén,K., Levin,M., Wiklund,O., Borén,J. and Hultén,L.M. (2012) Arachidonate 15-lipoxygenase type B knockdown leads to reduced lipid accumulation and inflammation in atherosclerosis. *PLoS One*, **7**, e43142.

15. Reith,W., LeibundGut-Landmann,S. and Waldburger,J.-M. (2005) Regulation of MHC class II gene expression by the class II transactivator. *Nat. Rev. Immunol.*, **5**, 793–806.

16. Paine,A., Kirchner,H., Immenschuh,S., Oelke,M., Blasczyk,R. and Eiz-Vesper,B. (2012) IL-2 Upregulates CD86 Expression on Human CD4+ and CD8+ T Cells. *J. Immunol.*, **188**, 1620–1629.

17. ZHA,H., SUN,H., LI,X., DUAN,L., LI,A., GU,Y., ZENG,Z., ZHAO,J., XIE,J., YUAN,S., *et al.* (2016) S100A8 facilitates the migration of colorectal cancer cells through regulating macrophages in the inflammatory microenvironment. *Oncol. Rep.*, **36**, 279–290.

18. Zhou,Z., Peng,Y., Wu,X., Meng,S., Yu,W., Zhao,J., Zhang,H., Wang,J. and Li,W. (2019) CCL18 secreted from M2 macrophages promotes migration and invasion via the PI3K/Akt pathway in gallbladder cancer. *Cell. Oncol. (Dordr).*, **42**, 81–92.

19. O'Shea,N.R., Chew,T.S., Dunne,J., Marnane,R., Nedjat-Shokouhi,B., Smith,P.J., Bloom,S.L., Smith,A.M. and Segal,A.W. (2016) Critical Role of the Disintegrin Metalloprotease ADAM-like Decysin-1 [ADAMDEC1] for Intestinal Immunity and Inflammation. *J. Crohns. Colitis*, **10**, 1417–1427.

20. Inazaki,K., Kanamaru,Y., Kojima,Y., Sueyoshi,N., Okumura,K., Kaneko,K., Yamashiro,Y., Ogawa,H. and Nakao,A. (2004) Smad3 deficiency attenuates renal fibrosis, inflammation,and apoptosis after unilateral ureteral obstruction. *Kidney Int.*, **66**, 597–604.

21. Li,Y., Zhang,P., Wang,C., Han,C., Meng,J., Liu,X., Xu,S., Li,N., Wang,Q., Shi,X., *et al.* (2013) Immune Responsive Gene 1 (IRG1) Promotes Endotoxin Tolerance by Increasing A20 Expression in Macrophages through Reactive Oxygen Species. *J. Biol. Chem.*, **288**, 16225–16234.

22. Fainaru,O., Woolf,E., Lotem,J., Yarmus,M., Brenner,O., Goldenberg,D., Negreanu,V., Bernstein,Y., Levanon,D., Jung,S., *et al.* (2004) Runx3 regulates mouse TGF-beta-mediated dendritic

cell function and its absence results in airway inflammation. *EMBO J.*, **23**, 969–79.

23. Hefetz-Sela,S., Stein,I., Klieger,Y., Porat,R., Sade-Feldman,M., Zreik,F., Nagler,A., Pappo,O., Quagliata,L., Dazert,E., *et al.* (2014) Acquisition of an immunosuppressive protumorigenic macrophage phenotype depending on c-Jun phosphorylation. *Proc. Natl. Acad. Sci.*, **111**, 17582–17587.
